# Particle and Phase Analysis of Combusted Iron Particles for Energy Storage and Release

**DOI:** 10.3390/ma16052009

**Published:** 2023-02-28

**Authors:** Simon Buchheiser, Max Philipp Deutschmann, Frank Rhein, Amanda Allmang, Michal Fedoryk, Björn Stelzner, Stefan Harth, Dimosthenis Trimis, Hermann Nirschl

**Affiliations:** 1 Process Machines, Institute of Mechanical Process Engineering and Mechanics, Karlsruhe Institute of Technology, 76131 Karlsruhe, Germany; 2 Combustion Technology, Engler-Bunte-Institute, Karlsruhe Institute of Technology, 76131 Karlsruhe, Germany

**Keywords:** particle characterization, metal fuels, iron combustion, nanoparticles, microexplosions, small-angle X-ray scattering (SAXS), wide-angle X-ray scattering (WAXS)

## Abstract

The combustion of metal fuels as energy carriers in a closed-cycle carbon-free process is a promising approach for reducing CO_2_ emissions in the energy sector. For a possible large-scale implementation, the influence of process conditions on particle properties and vice versa has to be well understood. In this study, the influence of different fuel–air equivalence ratios on particle morphology, size and degree of oxidation in an iron–air model burner is investigated by means of small- and wide-angle X-ray scattering, laser diffraction analysis and electron microscopy. The results show a decrease in median particle size and an increase in the degree of oxidation for leaner combustion conditions. The difference of 1.94 μm in median particle size between lean and rich conditions is twentyfold greater than the expected amount and can be connected to an increased intensity of microexplosions and nanoparticle formation for oxygen-rich atmospheres. Furthermore, the influence of the process conditions on the fuel usage efficiency is investigated, yielding efficiencies of up to 0.93. Furthermore, by choosing a suitable particle size range of 1 to 10 μm, the amount of residual iron content can be minimized. The results emphasize that particle size plays a key role in optimizing this process for the future.

## 1. Introduction

One major contributor to global warming is the production of energy by means of fossil fuels. Renewable alternatives such as solar or wind energy are dependent on weather conditions and fluctuate heavily. To attenuate these fluctuations, the development of carbon-free technologies for energy storage is necessary. Current approaches include pumped hydroelectric storage [1], Carnot batteries [2] or redox flow batteries [3]. However, these storage methods are regionally bound and offer limited options for transportation and trade. A promising alternative energy carrier is metal fuels [4], as they are suitable to be used in retrofitted, already-existing coal power plants [5]. The heat from the exothermic combustion of metal fuels into particulate metal oxides is then converted into electrical power. In contrast to carbon dioxide, the produced metal oxide in the exhaust can be easily separated for recycling using hydrogen from electrolysis. This grants the possibility of storing and releasing energy in large quantities in a carbon-free closed cycle. In essence, the reduction process (storage) could be performed in sun-rich, non-inhabited areas, whereas the oxidation process (release) yields the needed electrical energy in population-dense, industrial areas [4].

Various metal fuels can be used for combustion [6], such as aluminum [7], silicon [8,9] and iron. Iron particles offer multiple benefits as metal fuel: they are widely available, non-toxic and their boiling point is higher than their flame temperature [10]. The heterogenous combustion mode with air under atmospheric pressure prevents the iron particles and the formed products from being fully vaporized [11]. Thus, the produced iron oxide particles can be easily separated from the gas stream, e.g., via cyclonic separation. The following reduction process can be realized in a variety of industrial-scale technologies, ranging from the traditional blast furnace process to reduction with pure hydrogen produced by renewable electrical energy via the electrolysis of water. In the latter case, the cycle process emits nearly zero carbon into the atmosphere.

There are several options for lab-scale iron combustion: the oxidation process can be carried out with wall-heated laminar flow reactors [12] or iron dust flames with an additional [13] or without [10] an additional methane/air flame. In those experiments, particle size and morphology play a major role in the process conditions, just as in most metal-based reactions [14]. As the reaction kinetics is limited by surface diffusion [15], the necessary residence time may not be reached inside the reaction zone of the flame. Decreasing the particle size for better surface/volume ratios, however, leads to decreased flame temperatures [16]. For irregular or porous iron particles, the particles change their morphology to spheres during combustion due to melting [12]. Therefore, it can be assumed that particle size is more influential in combustion conditions than particle shape. Furthermore, in a closed-cycle process, minimal changes in particle morphology are strived for in order to keep all process conditions as steady as possible. Consequently, for the original iron particles, a spherical shape is ideal.

The occurrence of microexplosions in iron particles during combustion leads to breakage in primary particles and alters their size and morphology while simultaneously producing nanoparticles [17,18,19,20]. This phenomenon is suggested to be part of the formation mechanism of nanoparticles, though the formation of gaseous sub-oxides is another possibility [13]. Possible mechanisms include the diffusion of surrounding gases [21] and the vaporization of the unreacted core, as observed for aluminum [22], as well as the formation of iron pentacarbonyl due to residual carbon [17]. The small size of the nanoparticles increases the interparticle forces of the nanoparticles by orders of magnitude in relation to their gravitational force, leading to unwanted particle deposition and making energy-efficient separation more challenging. Furthermore, the cyclability of iron microparticles as energy carriers strongly depends on size preservation to grant steady process conditions.

Recent publications have focused on the detailed characterization of the combustion of iron particles [13,15,23] and in situ studies of nanoparticle formation [19,20,24]. However, an in-depth investigation of the changes in the particle properties of the combusted particles for different process conditions has not been reported yet. Hence, this work provides detailed information about the influence of the ratio of iron to oxygen on the distribution of different iron oxide phases, morphology aspects and the particle size distribution, as well as the resulting fuel usage efficiency. By studying the nanoparticle fractions with regard to their size distribution, further insights into already existing literature on the effect of microexplosions during combustion can be generated. A central aspect of this publication is the connection between particle properties and the envisioned cycle process for energy storage and release.

## 2. Materials and Methods

### 2.1. Experimental Setup

The combustion of the investigated powder took place in a tube burner utilizing an air-knife seeder for powder dispersion, which was discussed in more detail in a previous publication [25]. In this burner, a conical iron dust flame was generated, and the combusted particles were collected using a dedicated system.

### 2.2. Burner Setup

The iron powder for combustion was stored in a cylindrical tube with a diameter of 16.4 mm. It was pushed upward by a piston, the speed of which was controlled by a stepper motor connected to it via a threaded shaft. The dispersion of the dust took place in a circular gap with a height of approx. 30 µm, generating a high gas velocity. The mass flow of air in the seeder/burner and the co-flow stream was regulated by mass flow controllers on the feed lines.

After passing through the gap, the powder moved through a 45° constriction and a 5.6 mm inner diameter pilot tube before entering the 20.5 mm inner diameter combustion tube. As the iron powder suspension exits the pilot tube, the flow slows down, and the particles tend to adhere to the wall of the larger tube. The particle mass flow at the burner outlet was calculated based on the amount of powder supplied by the flask and the amount collected in the separate reservoir.

The outlet tube is surrounded by a 58.8 mm diameter co-flow to stabilize the flame at the burner rim and prevent external influences on the flame. In the experiments, the co-flow stream was fed with atmospheric air at a velocity matching the mean outlet velocity of the burner at 35 cm/s.

### 2.3. Particle Collection System

The burner is located under a 25 cm by 25 cm square hood made of stainless steel. It is connected to a 90° bend and a 90 cm long 1” stainless steel pipe. This section makes it possible to cool down the hot aerosol. Subsequently, the particles enter a commercial vacuum cleaner Dyson v10™ (Dyson Ltd., Singapore) for separation. In the initial separation stage, 14 cyclones separate most of the particles from the airflow. This fraction is collected in a bagless container and can be easily sampled for external investigation. After leaving the cyclone section, the air with the non-separated particles passes through two cloth filters, where the second, smaller fraction of combusted particles is collected.

### 2.4. Operating Conditions and Stoichiometry

Five different fuel–air equivalence ratios, $\Phi_{{\mathrm{Fe}}_{2}O_{3}}$, which are the ratios of the fuel-to-oxidizer ratio to the stoichiometric fuel-to-oxidizer ratio, were used in the experimental studies. It was assumed that iron(III) oxide is the final oxidation state of the combustion process for the definition of the ratios, as shown in Equation (1):

(1) $$4\;\mathrm{Fe}+3{\;O}_{2}\to 2{\;\mathrm{Fe}}_{2}O_{3}.$$

The stoichiometric mixture, according to Equation (1), corresponds to an air-to-fuel ratio (AFR) of 1.86 kg_Air_/kg_Fe_. In this case, the fuel-to-oxidizer ratio matches the stoichiometric value, and, therefore, the fuel–air equivalence ratio, $\Phi_{{\mathrm{Fe}}_{2}O_{3}}$, equals unity. Apart from that, two leaner (less fuel) and two richer (more fuel) mixtures listed in Table 1 were studied. 

All experiments were performed at least in duplicate, with the exception of $\Phi_{{\mathrm{Fe}}_{2}O_{3}}=1.0$, which was performed four times, and each sample was additionally measured in duplicate.

### 2.5. Iron Powder

**Figure 1:**
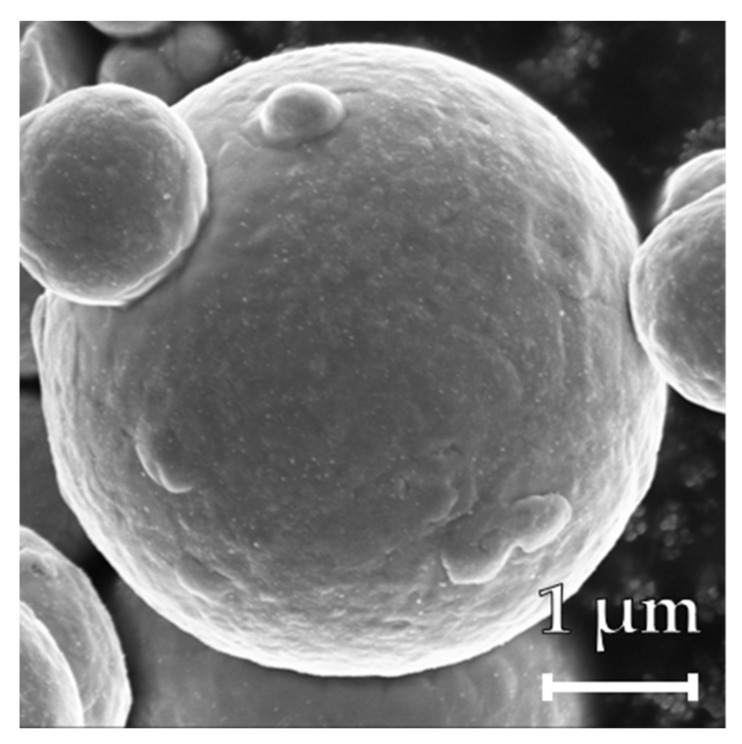
SEM image of the original carbonyl iron powder. The particles are spherical and non-porous.

The iron powder used in the combustion experiments was carbonyl iron powder (>99.5%, PMCtec GmbH, Leun, Germany). The SEM imaging in Figure 1 shows a spherical shape for the particles that is typical for carbonyl iron powders [26]. The median particle size is 5.67 μm, whereas a mass fraction of 1% is smaller than 0.9 μm, as measured with laser light diffraction. The iron powder was conditioned at 60 °C for a minimum of one day before each measurement to eliminate moisture and avoid particle clumping. The experiments, however, were performed at room temperature. The bulk density of the powder was found to be 2.98 g/cm^3^. Initial tests revealed powder loss in the outlet tube, which was calculated for all operating conditions [25]. The mass flow rate of the iron in the burner was determined by considering the displacement speed of the piston, the diameter of the dust reservoir, the bulk density of the powder and the powder losses in the outlet tube.

### 2.6. Particle Characterization

The particle size distributions (PSD) of the original iron and sampled iron oxide particles from the first separation stage were determined via laser diffraction analysis (HELOS (H0309) and QUIXEL, Sympatec GmbH, Clausthal-Zellerfeld, Germany). Additionally, the nanoparticles were investigated via SEM, and a comparative image analysis of these images with the software ImageJ [27] provided a number-based particle size distribution. The phase characterization of the iron particles was carried out by means of wide-angle X-ray scattering (WAXS). The measuring device was the Xeuss 2.0 Q-Xoom (Xenocs SA, Grenoble, France). The X-ray microfocus source was a Genix3D Cu ULC (Ultra Low divergence), which emits Cu-Kα radiation with an energy of 8.04 keV and a wavelength of 1.5406 Å. The scattered radiation was measured with a Pilatus3 R 300k detector (Dectris Ltd., Baden-Dättwil, Switzerland). The investigated powder samples were measured on adhesive polyimide foil with an exposure time of 30 min and a distance of 80 mm from the sample to the detector. Regarding nanoparticle analysis, the distance from the detector to the sample was changed to 2500 mm in order to detect small-angle X-ray scattering (SAXS). The combination of SAXS and WAXS measurements has the advantage of both providing detailed information about flame-born metal oxide nanoparticle sizes and morphology [28,29,30], as well as quantitative information about the degree of oxidation. 

#### 2.6.1. Calculation of the Weight Fractions of Iron and the Iron Oxide Phases in a Sample Using WAXS Calibration

**Figure 2:**
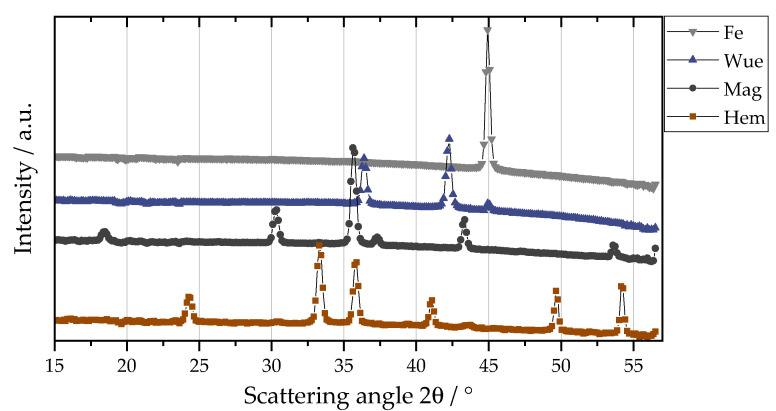
Characteristic Bragg peaks of the powder materials used for the phase quantification. The scattering curve for iron(II) oxide has an additional peak at 44.9° due to residual iron. The corresponding calibration curve was calculated with the assumption of 5% iron content, the maximum amount according to the producer.

The intensity ratio method for X-ray powder diffraction is a well-known method of quantitative phase analysis [31,32,33] that has also been adapted for WAXS. Scattering curves of multiple binary mixtures of pure materials are measured, and a linear regression model for peak intensity ratio as a function of the weight fraction ratio is determined. The pure materials used in this publication are carbonyl iron powder (>99.5%, PMCtec GmbH, Leun, Germany; see Section 2.5), iron(II) oxide (10 mesh; ≥99.6% trace metal basis, Sigma-Aldrich, St. Louis, MO, USA), iron(II,III) oxide (<5 μm, 95%, Sigma-Aldrich, St. Louis, MO, USA) and iron(III) oxide (<5 μm, 96%, Sigma-Aldrich, St. Louis, MO, USA). For better readability, the oxide states are abbreviated below with the corresponding mineral names, i.e., Fe (iron), Wue (wuestite, iron(II) oxide), Mag (magnetite, iron(II,III) oxide) and Hem (hematite, iron(III) oxide). In Figure 2 the wide-angle scattering curves of the pure materials are shown. The chosen characteristic Bragg reflection peaks of the materials are located at a scattering angle, 2θ, of 30.36° for iron(II,II) oxide (220) [34], 33.17° for iron(III) oxide (104) [35], 41.66° for iron(II) oxide (200) [36] and 44.69° for α-iron (110) [37]. The peaks were fitted with a Pseudo-Voigt profile using Profex 5.1.1 [38]. For a binary mixture of two materials, A and B, the relationship between the peak intensity ratio, I_A_/I_B_, and weight fraction ratio, X_A_/X_B_, is

(2) $$\frac{I_{A}}{I_{B}}=K_{\mathrm{AB}}\frac{X_{A}}{X_{B}}$$

with the proportionality constant, K_AB_. Therefore, plotting the intensity ratio of multiple mixtures with varying weight fraction ratios yields a linear plot with a y-intercept at 0 and a slope of K_AB_. As an internal standard, the Bragg peak of iron(III) oxide at 33.17° was chosen as it was present in all samples. The calibration plots and corresponding regression results for the mixtures of iron(II,III) oxide–iron(III) oxide (Mag-Hem), iron(II) oxide-iron(III) oxide (Wue-Hem) and iron–iron(III)–oxide (Fe-Hem) can be found in the Supplementary Information. The resulting proportionality factors are $K_{\mathrm{Mag}-\mathrm{Hem}}=0.440,{\;K}_{\mathrm{Wue}-\mathrm{Hem}}=0.583\;$ and $K_{\mathrm{Fe}-\mathrm{Hem}}=1.549$.

The mass ratios to the iron(III) oxide of an unknown mixture are then calculated by determining the intensity ratios and dividing them with the respective proportionality constant, K_AB_. Assuming mass closure, $X_{\mathrm{Mag}}+X_{\mathrm{Wue}}+X_{\mathrm{Fe}}+X_{\mathrm{Hem}}=1$, the mass fraction of iron(III) oxide is calculated according to

(3) $$X_{\mathrm{Hem}}=\frac{1}{1+K_{\mathrm{Mag}-\mathrm{Hem}}\;\frac{I_{\mathrm{Mag}}}{I_{\mathrm{Hem}}}+K_{\mathrm{Wue}-\mathrm{Hem}}\;\frac{I_{\mathrm{Wue}}}{I_{\mathrm{Hem}}}+K_{\mathrm{Fe}-\mathrm{Hem}}\;\frac{I_{\mathrm{Fe}}}{I_{\mathrm{Hem}}}\;}.$$

All other mass fractions can be determined using Equation (2) with the corresponding intensity ratio.

#### 2.6.2. Calculation of the Fuel Usage Efficiency

For the future use of iron powder in retrofitted coal power plants, the released amount of thermal energy during combustion is directly linked to the phase contents of the oxidized particles. An exemplary fuel usage efficiency, $\eta_{\mathrm{Hem}}$, was calculated from the relative phase contents as the ratio between the released energy during oxidation and the maximum possible released energy for full oxidation to ${\mathrm{Fe}}_{2}O_{3}$:

(4) $$\eta_{\mathrm{Hem}}=\frac{\Delta_{r}H_{\mathrm{calc},\mathrm{particle}}}{\Delta_{r}H_{\mathrm{calc},\max}}.$$

$\Delta_{r}H_{\mathrm{calc},\max}$ represents the maximum available reaction enthalpy per kg of product particles if the iron fraction was fully converted to iron(III) oxide. Using the weight fractions determined by WAXS calibration and the standard enthalpy of the formation of iron(III) oxide, the maximum available reaction enthalpy is provided by

(5) $$\Delta_{r}H_{\mathrm{calc},\max}=\frac{1}{2}\left(3\frac{X_{\mathrm{Mag}}}{{\tilde{M}}_{\mathrm{Mag}}}+2\frac{X_{Häm}}{{\tilde{M}}_{Häm}}+\frac{X_{\mathrm{Wue}}}{{\tilde{M}}_{\mathrm{Wue}}}+\frac{X_{\mathrm{Fe}}}{{\tilde{M}}_{\mathrm{Fe}}}\right)\Delta_{f}H_{Häm}.$$

On the other hand, $\Delta_{r}H_{\mathrm{calc},\mathrm{particle}}$ is the sum of the molar fractions of each oxide phase after multiplying it with its respective standard enthalpy of formation. The assumed enthalpies were −272.04 kJ/mol for iron(II) oxide, −1120.89 kJ/mol for iron(II,III) oxide and −825.5 kJ/mol for iron(III) oxide [39].

#### 2.6.3. Small-Angle X-ray Scattering

Nanoparticles scatter incoming X-rays characteristically depending on their nanoscale structures, such as particle size and morphology. The resulting scattering curve is a double logarithmic plot of the intensity, I, over the scattering vector, q, in Å^−1^. The scattering vector, q, in Equation (6) describes the scattering angle, 2θ, independent of the wavelength, λ, of the primary beam and is provided by

(6) $$q=\frac{4\pi }{\lambda }\sin\left(\theta \right).$$

Information about particle size is retrieved with Guinier’s law in Equation (7) at small angles (qR_g_ < 1) as the radius of gyration, $R_{g}$, with prefactor G:

(7) $$I\left(q\right)=G\exp\left(-\frac{q^{2}R_{g}^{2}}{3}\right).$$

The slope of the scattering curve is provided by a local power-law fit with prefactor B according to

(8) $$I\left(q\right)={B\;q}^{-p}.$$

If the exponent, P, for high scattering vectors equals 4, Porod’s law for smooth particles with a sharp density transition is fulfilled [40]. The Unified Fit Model (Irena Package 2.71 [41], IgorPro, Wavemetrics), according to Beaucage [42], was used to evaluate the scattering data. Assuming spherical primary particles with log-normal size distribution the geometric standard deviation, $\sigma_{g}$ is calculated with the polydispersity index (PDI) and the parameters of Guinier’s law and the power-law fit [28]:

(9) $$\sigma_{g}=\exp\left(\sqrt{\frac{\ln\left(\mathrm{PDI}\right)}{12}}\right);\;\mathrm{PDI}=\frac{{B\;R}_{g}^{4}}{1.62\;G}.$$

The median diameter, d_SAXS_, of the distribution is then provided by [43]

(10) $$d_{\mathrm{SAXS}}=2\sqrt{\frac{5}{3}}R_{g}\exp\left(-13\frac{\ln\left(\mathrm{PDI}\right)}{24}\right).$$

## 3. Results

**Figure 3:**
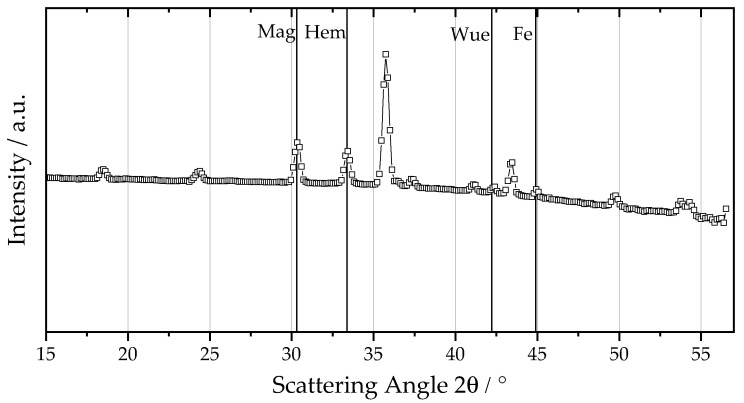
Wide-angle scattering curve of an iron oxide sample produced with a fuel–air equivalence ratio of $\Phi_{{\mathrm{Fe}}_{2}O_{3}}$ = 1.5. The particle exhibits four characteristic Bragg peaks due to the respective iron and iron oxide phases: α-iron (Fe) at 44.9°, iron(II) oxide (Wue) at 42.2°, iron(II,III) oxide (Mag) at 30.3° and iron(III) oxide (Hem) at 33.4°.

**Figure 4:**
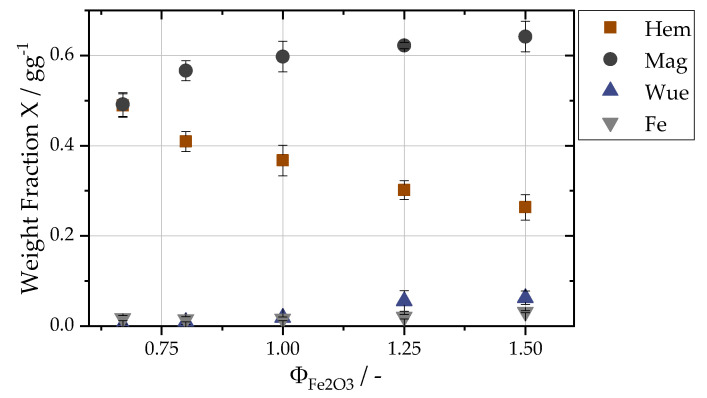
Weight fractions of the respective oxide phases over $\Phi_{{\mathrm{Fe}}_{2}O_{3}}$, as calculated with WAXS calibration (Section 2.6.1).

Figure 3 shows an exemplary WAXS curve of oxidized particles from the first separation stage for a fuel–air equivalence ratio of 1.5. All characteristic Bragg peaks can be assigned respectively to iron(III) oxide, iron(II,III) oxide, iron(II) oxide or α-iron, showing that the sampled iron oxide particles consist only of various iron oxide and pure iron phases. Based on the relative intensity ratios, the relative phase contents have been calculated using the WAXS-calibration method. The results of increasing the fuel–air equivalence ratios. $\Phi_{{\mathrm{Fe}}_{2}O_{3}}$, are depicted in Figure 4. The iron(III) oxide content decreases steadily from 0.49 ± 0.03 to 0.26 ± 0.03 for richer mixtures, whereas the iron(II,III) oxide content increases from 0.49 ± 0.03 to 0.64 ± 0.03. The residual iron content is highest for fuel-rich flames with 0.03 and then decreases to values of around 0.016. The steady increase for the higher oxidized phases with decreasing equivalence ratios, $\Phi_{{\mathrm{Fe}}_{2}O_{3}}$, shows that lean flames yield a better degree of oxidation.

**Figure 5:**
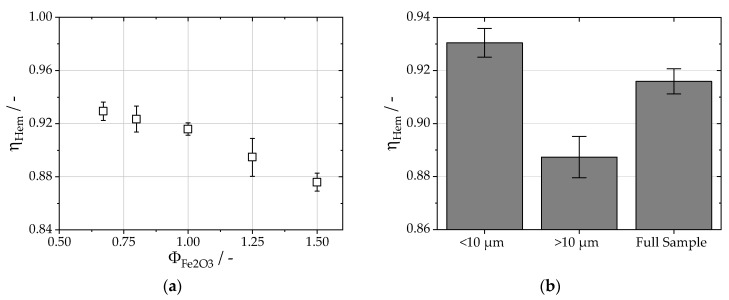
(**a**) Fuel usage efficiency for different fuel–air equivalence ratios. The efficiency is calculated as the ratio between the released energy during oxidation and the maximum possible released energy for full oxidation into Fe_2_O_3_. (**b**) Fuel usage efficiency for the stoichiometric ratio of oxygen to iron particles. The particles have been separated into two fractions, one above and one below 10 μm. The larger fraction shows increased residual iron content, which leads to decreased fuel usage efficiency.

The fuel usage efficiency, $\eta_{\mathrm{Hem}}$, is provided in Figure 5a for varying equivalence ratios, $\Phi_{{\mathrm{Fe}}_{2}O_{3}}$. For $\Phi_{{\mathrm{Fe}}_{2}O_{3}}=1.5$, $\eta_{\mathrm{Hem}}$ is lowest with 0.87 ± 0.01 and rises with decreasing fuel–air equivalence ratios. The changes between $\Phi_{{\mathrm{Fe}}_{2}O_{3}}=1.0$ and 0.67 become negligible with $\eta_{\mathrm{Hem}}$, ranging from 0.91 ± 0.01 to 0.93 ± 0.01. To put these values in perspective, a full conversion of pure iron powder to pure iron(II) oxide results in $\eta_{\mathrm{Hem}}$ = 0.66, and a conversion to pure iron(II,III) results in $\eta_{\mathrm{Hem}}$ = 0.91. These results show that minimizing the residual iron content is of utmost importance since a large portion of the energy is already released after the first oxidization stage into iron(II). To understand the effect of particle size on the degree of oxidation, the sampled iron oxide particles for $\Phi_{{\mathrm{Fe}}_{2}O_{3}}=1.0$ were sieved into two fractions for particles smaller and larger than 10 μm. The direct comparison of the fuel usage efficiency for the two fractions with the original sample is shown in Figure 5b. Due to the higher weight fraction of residual iron (0.016) and iron(II) oxide (0.048), $\eta_{\mathrm{Hem}}$ decreases to 0.89 ± 0.01 for the coarse fraction. The fine fraction, on the other hand, has a slightly increased $\eta_{\mathrm{Hem}}$ at 0.93 ± 0.01 compared with the original sampled particles ($\eta_{\mathrm{Hem}}$ = 0.92 ± 0.01). The ratio of the available surface for oxidation and the volume of the particle decreases with increasing particle size. Therefore, larger particles are more difficult to oxidize completely. Based on the fractionation, the target product particle size is 10 μm. Knowing the respective densities of iron(III) oxide and α-iron, the resulting maximum particle size of the carbonyl iron powder should be below 7 μm to achieve almost full oxidation.

**Figure 6:**
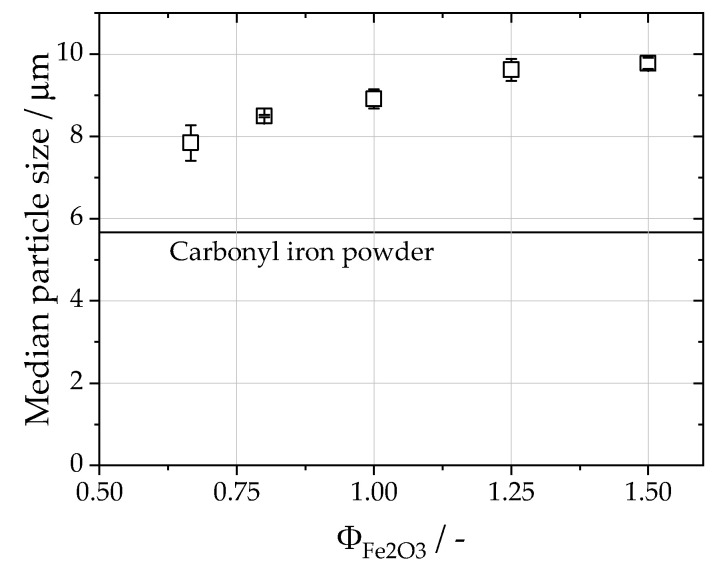
Median particle size of the iron oxide particles for different fuel–air equivalence ratios, $\Phi_{{\mathrm{Fe}}_{2}O_{3}}$. The median of the carbonyl iron powder is included as a straight line for reference.

**Figure 7:**
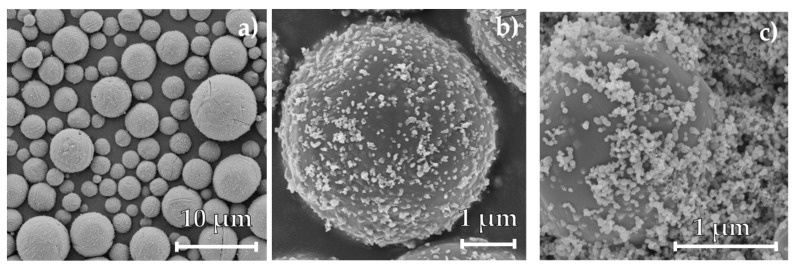
SEM images of the sampled iron oxide particles after combustion at different resolutions, (**a**) and (**b**), in the first separation stage and (**c**) in the second separation stage for a fuel–air equivalence ratio of 1. The particles in the first separation stage are spherical, exhibit multiple cracks and holes on the surface and are covered by fine nanoparticles adhering to the surface. In (**c**), the separated nanoparticles show a smooth surface as well as a spherical morphology.

Particle size distributions of all collected samples in the first separation stage were measured, and the corresponding median values are shown in Figure 6, while the median of the original iron powder is plotted as a horizontal line. The full PSDs are available in the Supplementary Material. After oxidation, the median particle size increases from 5.67 ± 0.45 μm to sizes between 9.78 ± 0.13 μm and 7.84 ± 0.44 μm due to the embedded oxygen inside the particles, resulting in a decrease in density from 7800 kg/m^3^ for pure iron to 5201 kg/m^3^ for iron(II,III) oxide and 5865 kg/m^3^ for iron(II) oxide at an ambient temperature [44]. The density of iron(III) oxide with 5277 kg/m^3^ is roughly equal to the density of iron(II,III) oxide. With the weight fractions and densities of the oxide phases, as well as the median particle size of 5.67 μm for the carbonyl iron particles, an exemplary median particle size for the particles after combustion of 8.43 μm for $\Phi_{{\mathrm{Fe}}_{2}O_{3}}=0.67$ and 8.35 μm for $\Phi_{{\mathrm{Fe}}_{2}O_{3}}=1.5$ was calculated. However, the difference in the particle size of the two respective fuel–air equivalence ratios measured is 1.94 μm, twentyfold larger than the calculated difference. In Figure 7, SEM images of the sampled iron oxide are depicted. The sampled iron oxide particles have a spherical morphology and are covered by nanoparticles. A fraction of the sampled iron oxide particles shows cracks along the particle surface. Neither the cracks nor the nanoparticles were apparent before combustion (see Figure 1). During combustion, bubble growth and the coalescence of gaseous phases lead to a pressure buildup inside the molten particles [10,21]. The pressure then breaks up the particle surface, and the gases are released. The microexplosion leads to the creation of nanoparticles that adhere to the surface and the remaining cavity of the oxidized particles. Furthermore, the released droplets of the molten core solidify as iron oxide particles. Therefore, the decreasing particle size for increasing oxygen content can be linked to an increase in microexplosions and nanoparticle production.

**Figure 8:**
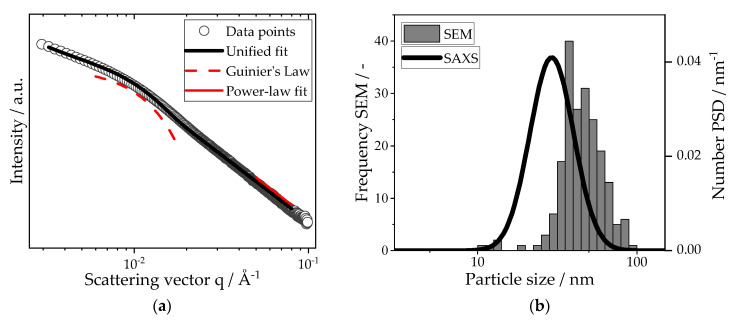
(**a**) Small-angle X-ray scattering curve of the iron oxide nanoparticles. The original scattering data have been fitted (with local Guinier and power-law fits) using a unified fit approach. Guinier’s law yields a prefactor of 413.27 with a radius of gyration of 22.6 nm. The evaluation of the power-law fit delivers an exponent of −4 and a prefactor of 5.58·10^−7^. (**b**) Particle size distributions of the nanoparticles. Both distributions have a geometrical standard deviation of 1.37. For the SEM distribution, 300 particles have been counted.

The SAXS scattering curve of the produced nanoparticles, as separated by the cloth filter, with the accompanying unified fit as well as the local Guinier and power-law fits, is depicted in Figure 8a, yielding a radius of gyration of $R_{g}=22.6\;\mathrm{nm}$ and an exponent, P, of −4. The power law supports the drawn assumption of spherical particles with a smooth surface. Using the method described by Beaucage et al. [28], particle size distributions with the assumption of log-normal distributed spheres were derived from the fit parameters of the scattering curve. Figure 8b shows the direct comparison of this PSD with the PSD obtained from SEM imaging. The median particle size of the SAXS distribution is 32.3 nm, whereas the median particle size of the SEM image analysis is 44.1 nm. Small differences in absolute values come about due to resolution limits for SEM imaging, as well as possible deviations from the drawn assumptions of the SAXS data. However, both values are in the same order of magnitude and emphasize the production of nanoparticles during combustion.

## 4. Discussion

The investigated combustion experiment provides valuable information on the use of iron as energy carrier, especially with regard to changes in its particle sizes and morphology. Iron particles were oxidized up to an oxygen mass fraction of 0.283 (compared with an oxygen mass fraction of 0.3 for the full oxidation into iron(III) oxide) with a mass fraction of residual iron of less than 0.02. The degree of oxidation was raised for leaner mixtures by decreasing the fuel–air equivalence ratios. However, our findings show that microexplosions occur during combustion, which increase in intensity for decreased $\Phi_{{\mathrm{Fe}}_{2}O_{3}}$, ultimately leading to increased nanoparticle formation in oxygen-rich atmospheres. These results are supported by the findings of Huang et al. [24] as well as Wright et al. [45], who also report favorable conditions for microexplosions in an oxygen-rich atmosphere. The other mechanism of nanoparticle formation is the condensation of iron-containing gaseous species, which also increase in intensity for higher oxygen contents in the flame [46]. Both effects may be present simultaneously, and future studies should, therefore, select their equivalence ratios with respect to the process conditions. The nanoparticles are spherical with a median diameter of 32 nm and a smooth surface, and they adhere to the surface and cavities of the sampled iron oxide particles. The data are in good agreement with previously reported findings [12]. 

The calculated fuel usage efficiencies increase from 0.88 to 0.93 for leaner atmospheres with decreasing fuel–air equivalence ratios. The main influence to ensure high efficiency is minimizing residual iron content in the oxidized particles. Particle size was also identified to have a significant effect, as larger fractions were oxidized to a lesser degree than smaller fractions. To achieve almost full oxidation, the maximum particle diameter of the carbonyl iron particles should, therefore, be limited. Furthermore, Li et al. [17] reported an increased frequency of microexplosions for larger particles of up to 100 μm, providing an additional reason to keep particle sizes small. On the other hand, the minimum particle size should also be limited due to an increased risk of spontaneous ignition [47], increased effort for separation [48] and general challenges in solids handling, e.g., agglomeration, low packing density and powder flowability.

Generally, the microexplosion mechanism was shown to drastically change the particle size and morphology and is, therefore, crucial to the applicability of iron particles in a circular energy storage system. Therefore, further research should focus on identifying the dependency of microexplosions on the process conditions and finding ways to inhibit microexplosions while retaining a good degree of oxidation with minimal residual iron content. Our findings suggest that there is an optimal air-to-fuel ratio (in our study, around 2 kg_Air_/kg_Fe_ for a median carbonyl iron powder particle size of 5.67 μm), as the sampled combustion product was well oxidized, and the nanoparticle production was reduced compared with higher air-to-fuel ratios.

This publication cements the applicability of iron particles as a metal fuel for energy storage and release in retrofitted powerplants. For the envisioned cycle process, high fuel usage efficiencies while retaining a similar morphology and particle size are desired. Therefore, the influence of particle properties on the process conditions and vice versa will be a central aspect of future studies in order to optimize the process.

## Figures and Tables

**Table 1:** Investigated experimental conditions.

AFR	kg_Air_/kg_Fe_	1.24	1.49	1.86	2.33	2.79
$\Phi_{{\mathrm{Fe}}_{2}O_{3}}$	-	1.50	1.25	1.00	0.80	0.67

## Data Availability

The data presented in this study are available on request.

## Supplementary Materials

The following supporting information can be downloaded at: https://www.mdpi.com/article/10.3390/ma16052009/s1.
