# Supplementary material for: Particle and Phase Analysis of Combusted Iron Particles for Energy Storage and Release
Source: Materials (Basel). 2023 Feb 28;16(5):2009. doi: 10.3390/ma16052009 (PMC10004356; doi:10.3390/ma16052009)
Supplement: Supplementary file 1 [file materials-16-02009-s001.zip › materials-2251981-supplementary.pdf]

materials

Article

Particle and Phase Analysis of Combusted Iron Particles for Energy Storage and Release

Simon Buchheiser [1]*, Max Philipp Deutschmann [1], Frank Rhein [1], Amanda Allmang [1], Michal Fedoryk [2], Björn Stelzner [2], Stefan Harth [2], Dimosthenis Trimis [2] and Hermann Nirschl [1]

[1] Karlsruhe Institute of Technology, Institute of Mechanical Process Engineering and Mechanics, Process Machines, 76131 Karlsruhe, Germany
2 Karlsruhe Institute of Technology, Engler-Bunte-Institute, Combustion Technology, 76131 Karlsruhe, Germany
* Correspondence: simon.buchheiser@kit.edu (S. B.)

Supplementary information:

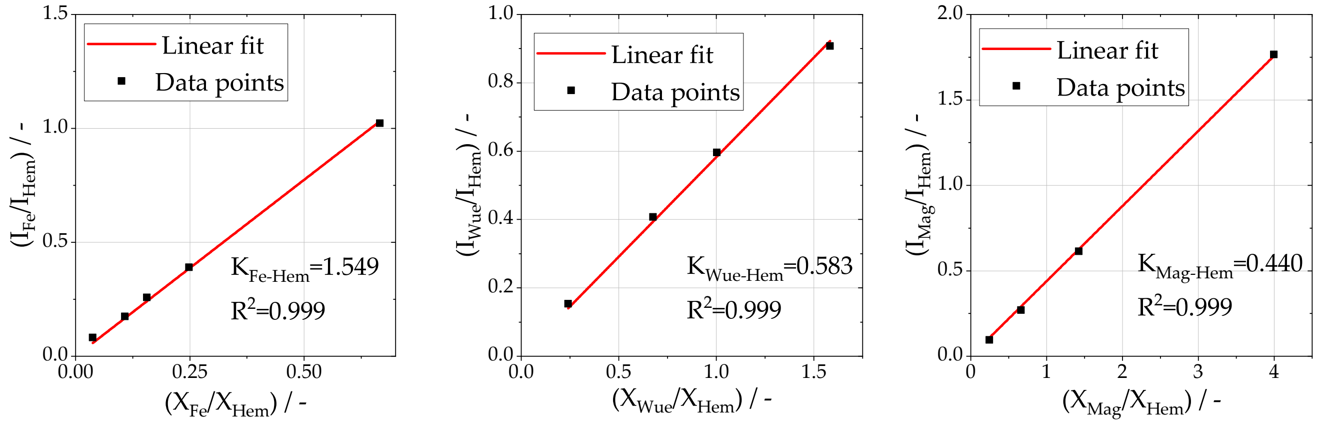

(**a**) (**b**) (**c**)

Supplementary S1. WAXS calibration lines for (**a**) iron - iron(III)oxide (Fe-Hem), (**b**) iron(II) oxide – iron(III) oxide (Wue-Hem) and (**c**) iron(II,III) oxide – iron(III) oxide (Mag-Hem).

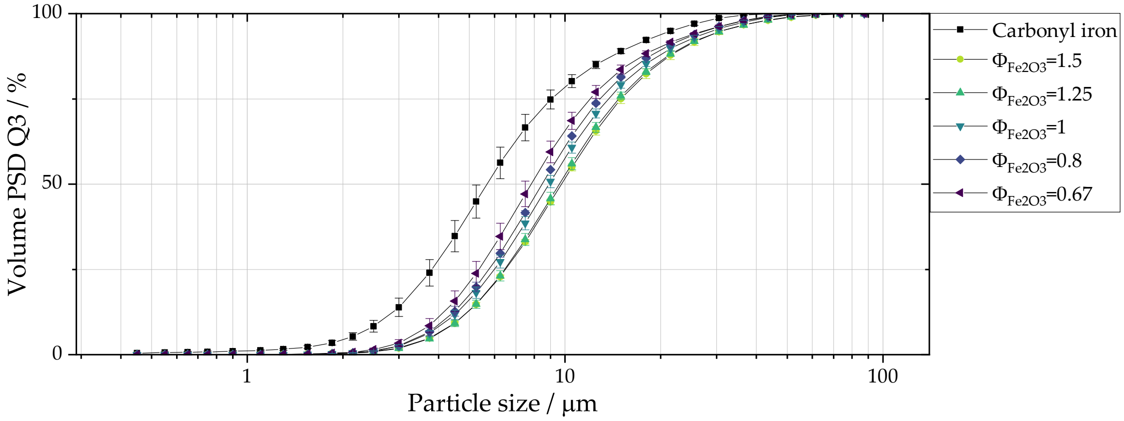

Supplementary S2. Particle size distributions as derived out of laser light diffraction analysis.

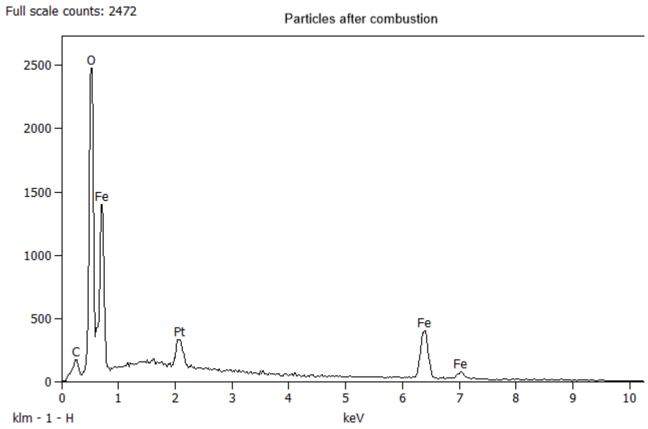

(**a**)

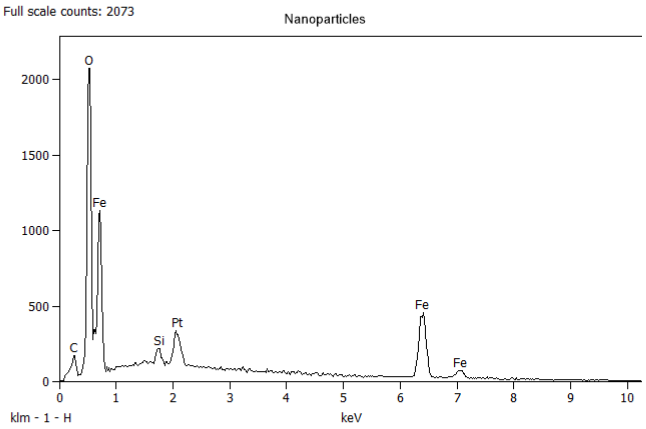

(**b**)

Supplementary S3. Elemental maps corresponding to fig. 7. In (**a**) the elemental map for the particles after combustion are depicted, whereas in (**b**) the map corresponding to the nanoparticulate fraction is shown. Both show qualitative information about the degree of oxidation on the particle surface and further validate the high oxygen fraction as calculated with the WAXS calibration.
